# Hematologic and Clinical Aspects of Experimental Ovine Anaplasmosis Caused by *Anaplasma ovis* in Iran

**Published:** 2012

**Authors:** SP Yasini, Z Khaki, S Rahbari, B Kazemi, J Salar Amoli, A Gharabaghi, SM Jalali

**Affiliations:** 1 Department of Clinical Pathology, Faculty of Veterinary Medicine, University of Tehran, Tehran, Iran; 2 Parasitology Department, Faculty of Veterinary Medicine, University of Tehran, Tehran, Iran; 3 Cellular and Molecular Biology Research Center, Shahid Beheshti University of Medical Sciences, Tehran, Iran; 4 Department of Toxicology, Faculty of Veterinary Medicine, University of Tehran, Tehran, Iran; 5 Department of Larg Animal Internal Medicine, Faculty of Veterinary Medicine, University of Tehran, Tehran, Iran

**Keywords:** *Anaplasma ovis*, Sheep, Experimental, Anemia, Iran

## Abstract

**Background:**

*Anaplasma ovis* infections can cause clinical symptoms in acute phase and lead to huge economic losses in flocks. The aim of the present study was to investigate the hematological and parasitological changes in experimental anaplasmosis in sheep with Iranian strain of *A. ovis*.

**Method:**

Five male sheep without any blood parasite infection were selected. One hundred ml heparinized blood was collected from splenectomised sheep that showed 6% *A. ovis* parasitemia. Inoculums of 20 ml blood were administered intravenously to each test animal. Hematological, parasitological and clinical changes of experimental anaplasmosis were studied in 0-38 days post infection.

**Result:**

Parasitemia was detected 3 days post infection and reached its maximum level on the day 12 of experiment in test animals. Then the parasitemia was declined, but the organism could be found persistently until the last day of study. The red cell counts, packed cell volume and hemoglobin concentration were decreased and mean corpuscular volume was increased significantly during the infection period. Reticulocytosis and basophilic stippling were also detected. No significant changes were observed in total and differential leukocyte count and animal body temperature.

**Conclusion:**

Experimental *A. ovis* infection in sheep resulted in marked normocytic normochromic anemia at the beginning of the infection which became macrocytic normochromic by the development of the disease. There were negative correlations between parasitemia and RBC, PCV and Hb values, therefore hematological assessment can be considered as a practical diagnostic tool in ovine anaplasmosis.

## Introduction

*Anaplasma ovis* is an intraerythrocytic rickettsial pathogen of sheep, goats and wild ruminants (1–5).The acute phase of the disease is characterized by severe anemia, fever, weight loss, abortion, lower milk production, pallor of mucous membrane, jaundice and often death (6, 7). Anaplasmosis is endemic in tropical and subtropical areas but is frequently reported in temperate regions of the world (7–9). Ovine anaplasmosis is mainly caused by *A. ovis* and *A. marginale*. Anaplasmosis is transmitted by ticks. Biting insects or inoculation of blood into susceptible animals can also transmit the disease (10, 11). Recovered animals become carriers. Splenectomy and intercurrent infections lower the resistance of the animals and render them more susceptible to the disease. Other stress factors such as malnutrition and pregnancy also increase the susceptibility of animals to anaplasmosis. During the acute stage of the disease, a diagnosis in the individual animal is usually made on the basis of clinical signs, the presence of the organism in blood smear and hematological evidence of infection (12).

Although some pathogenic aspects of anaplasmosis have been investigated before, but still this disease is a matter of importance for the researchers, thus the aim of this study was to evaluate the hematological, parasitological and clinical changes of experimental anaplasmosis in sheep with Iranian strain of *A. ovis* based on microscopic and molecular identification.

## Material and Methods

### Source of experimental animals

Twenty local breed sheep, about 5-6 month old were selected from a farm in Varamin, Tehran Province, Iran. The blood samples were collected in vacutainer tubes containing EDTA.

### Microscopic examination

The collected blood samples were used to prepare thin blood smears for microscopic examination. Blood smears were fixed with methanol for five min, stained with Giemsa at a dilution of 5% in buffer solution for 30 min, and then examined for the presence of blood parasite such as *Anaplasma, Theileria* and *Babesia* spp. under oil immersion lens (100×). Blood smears were recorded as negative for all hemoparasites if no inclusion bodies were observed in a 200-oil-immersion field.

### PCR analysis

DNA extraction was performed by using molecular biological system transfer kit (MBST Iran), based on the manufacturer's instructions. A PCR method was carried to detect *Anaplasma* spp. (*A. ovis* and *A. marginale*) based on the msp4 gene sequence and they were differentiated from each other by PCR-RFLP using HpaII enzyme similar to the method of Ahmadi-Hamedani and Khaki et al. (13).

*Theileria* and *Babesia* infection was ruled out by PCR technique using specific primers for 18SrRNA gene (14).

### Selection of animals

Five male sheep were selected from above as recipient animals for the experimental study. All selected animals were tested negative for the infection of blood parasites microscopically which were confirmed by PCR method.

An *A. ovis* infected sheep with no other blood parasites based on blood smear and PCR examination, was selected as donor and splenectomised to induce high level of parasitemia.

The selected animals were purchased and transferred to Veterinary Research and Teaching Hospital, Faculty of Veterinary Medicine, University of Tehran. All animals were treated against internal and external parasites14 days prior to the study.

### Experimental infection

The donor was subjected to blood collection for inoculation, three weeks after splenectomy while showing 6% *A. ovis* parasitemia. One hundred ml blood was collected in container with heparin anticoagulant from donor and an inoculum of 20 ml blood was administered intravenously to each test animal.

### Clinical examination and Sampling

Two ml of blood sample was collected via the jugular vein into EDTA from each test animal prior inoculation (day 0) and weekly thereafter (days 3, 5, 8, 9, 12, 15, 18, 20, 23, 25, 27, 30, 32, 34, and 38 post inoculation) for hematological and parasitological analysis. Physical examination was performed and clinical signs including rectal temperature, anorexia, depression and color of the mucous membranes (e.g., conjunctival and oral) were recorded on each animal.

### Microscopic examination

Blood smears were prepared and fixed with methanol for 5 min and stained with 5% Giemsa solution for 30 min and then examined for the presence of *Anaplasma* inclusion bodies under oil immersion lens (100×).

Parasitemia ratio was assessed by counting the number of infected red blood cells on examination of at least 200 microscopic fields. The number of infected cells was then expressed as a percentage.

Reticulocyte and basophilic stippling percentage was calculated by counting them per 1000 red cells in cresyl blue and Giemsa stained blood smears respectively.

### Hematological assessment

Hematological parameters included, total erythrocyte count (RBC), packed cell volume (PCV), hemoglobin concentration (Hb), mean corpuscular volume (MCV), mean corpuscular hemoglobin (MCH), mean corpuscular hemoglobin concentration (MCHC), total white blood cells (WBC) and differential leukocyte count were estimated as described by Meyer and Harvey (2004) (15).

### Statistical analysis

Analysis of variance (ANOVA) and Tukey tests were used for statistical differences between the groups. All values were expressed as mean ± standard error of mean (SEM) and *P*<0.05 was considered as statistically significant.

## Result

### Parasitological findings

**Fig. 1:**
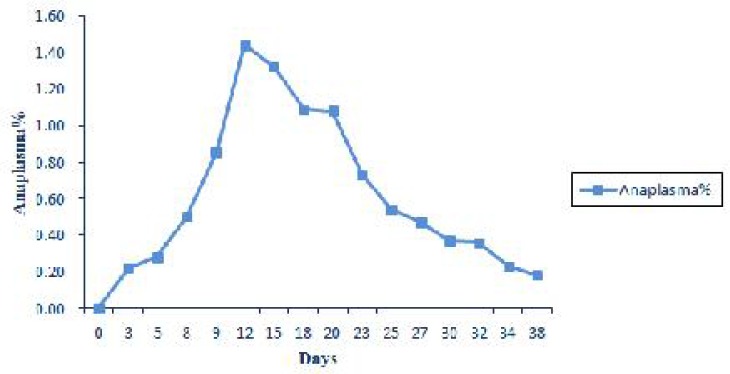
Expression of changes in prasitemia during experimental period

**Fig. 2:**
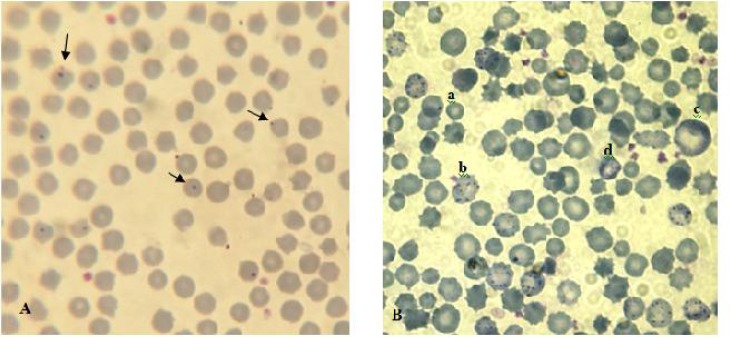
*Anaplasma ovis* infected sheep blood smear. A: *Anaplasma* inclusion body. B: a. *Anaplasma ovis* inclusion body, b: Basophilic stippling, c: Macrocytic RBC,d: polychromatophilic RBC

Microscopic examination of blood smears obtained from test animals revealed that the percentage of *Anaplasma* inclusion bodies increased significantly with the progress of infection and reached a peak of 1.44 ± 0.46% on the day 12 of experiment. Then, from this point to day 38, there was a gradual decline in parasitemia percentage to a minimum of 0.18 ± 0.03% (Fig. 1). In infected sheep erythrocytes 62.5-86% (Mean. 74.53%) of the inclusion bodies were located centrally or submarginally and the remaining 14-37.5% (Mean. 25.47%) were situated marginally (Fig. 2A).

### Hematologic and clinical changes

**Fig. 3:**
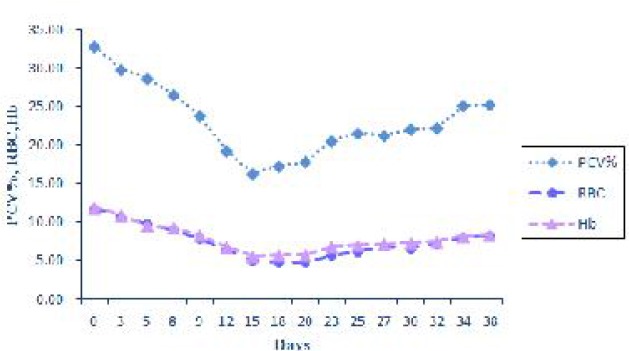
Expression of changes in PCV, RBC and Hb during experimental period

The data showed that the values of PCV, RBC and Hb decreased soon after the appearance of parasitaemia, reaching their lowest levels a few days after the peak of parasitaemia. After that there was a slight rise in the amount of these parameters until the last day of experiment (Fig. 3).

**Fig. 4:**
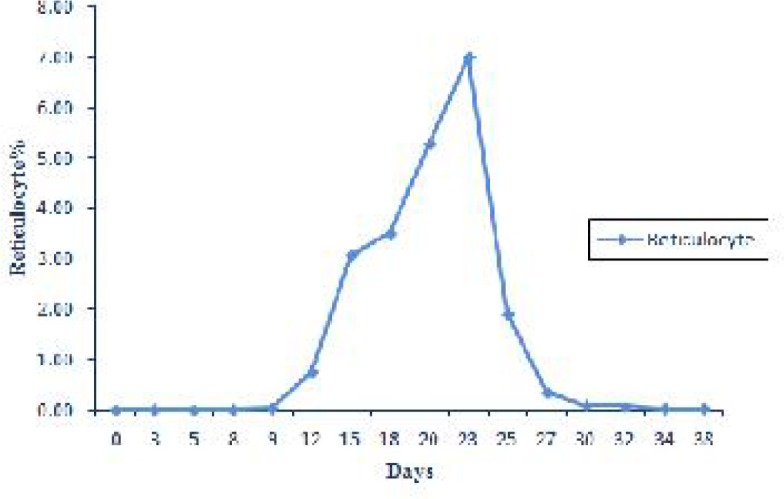
Expression of changes in reticulocyte % during experimental period

Reticulocytes count started to grow from 0.002 ± 0.002% on the day 8 to a high of 7.00 ± 3.76% on the day 23 post-inoculation followed by a downward trend to the end of study (Fig. 4).

**Fig. 5:**
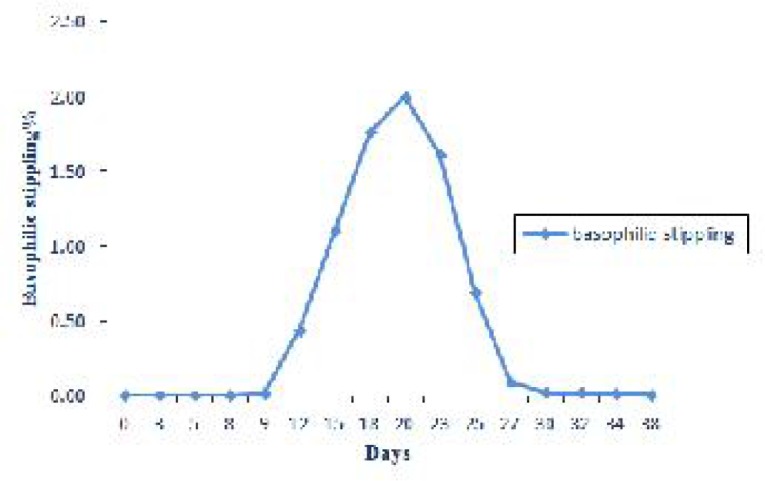
Expression of changes in basophilic stippling % during experimental period

Basophilic stippling was detected microscopically 9 days post infection and reached a peak 2.00 ± 0.32 on the day 20 of experiment (Fig. 5 and 2B).

**Fig. 6:**
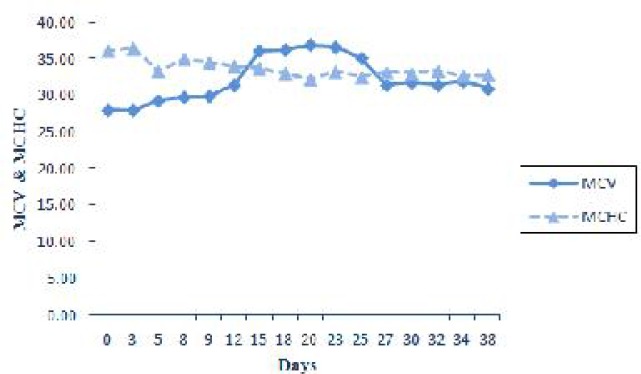
Expression of changes in MCV and MCHC during experimental period

A significant increase in MCV was observed coincidentally with the rise of reticulocyte on the day 20 and 23 post infection. There was no significant difference in MCHC. This indicates the development of regenerative macrocytic normochromic anemia at this stage of study, but at the beginning of the experiment the anemia was nonregenerative by normocytic normochromic appearance (Fig. 6).

Polychromasia was observed from the day 12 and increased gradually until day 20 which then returned to almost 0% by the end of experiment.

**Fig. 7:**
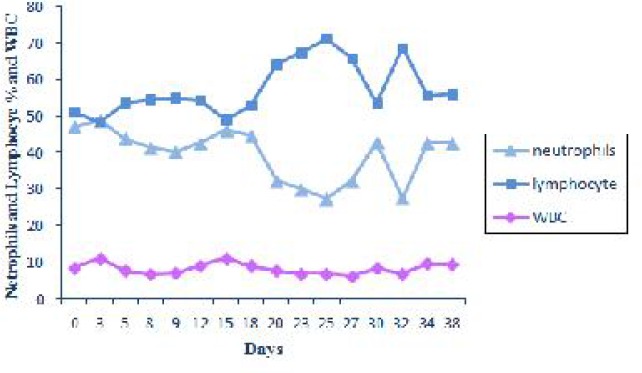
Expression of changes in neurophils and lymphocytes % and WBC during experimental period

In comparison with the first day, neutrophils percentage decreased between 5-38 days post infection and it reached its minimum level on the day 25 post infection. The reverse pattern was observed in lymphocytes percentage. However these changes were not significant. There was also no significant difference in the mean of WBC count (Fig. 7).

**Fig. 8:**
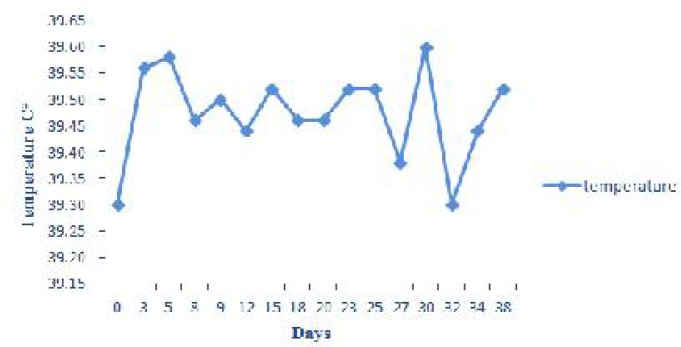
Expression of changes in temperature during experimental period

In our experiment during the days 3-38 post infection, body temperature over 39.5 was seen only in one or two sheep each day, thus no significant changes in mean body temperature were observed in statistical analysis (Fig. 8).

The mucus membranes were anemic on the day 12-23 post infection without any evidence of clinical icterus. During these days infected animals showed varying degrees of anorexia and listlessness.

## Discussion

Studies of experimental ovine anaplasmosis are very scarce in Iran and little information had been provided. This preliminary study was done for the first time in Iran. Our experiment indicated that parasitemia increased significantly along with the progress of infection and displayed its highest level 12 days post infection. The parasitemia reached to maximum level one or two weeks after first parasites detectable in the blood smear of infected animals (12). The parasitaemia then diminished slowly, but the organism could constantly be found until observations were stopped 38 days post infection. Decline parasite numbers in nonsplenectomised sheep, may be due to removal the infected erythrocyte from the circulation by reticuloendothelial system (16).

A few days after the peak of parasitemia, progressive anemia was appeared, in which the values of Hb, RBC and PCV gradually decreased from 3 days post inoculation and reached approximately their lowest levels on the day 15 post infection. In our experiment there was no evidence of intravascular hemolysis, therefore anemia occurred as a result of phagocytosis of infected erythrocytes to removal parasites during the period of rising parasitemia. Hemoglobinuria was an unusual clinical sign of anaplasmosis, because anemia results from extravascular opsonization and phagocytosis of parasitized erythrocytes by reticuloendothelial cells (17). However the severity of anemia may be as a result of immune-mediated destruction of non parasitized erythrocytes in addition to that of parasitized (18).

We observed negative correlations (*P*<0.01) among parasitemia and RBC count (r=−0.494), Hb (r=−0.496) and PCV (r=−0.570). The anemia increased as the severity of parasitemia increased, as Nazifi et al. (19) noted previously in cattle anaplasmosis.

In our experiment in infected sheep erythrocytes 62.5-86% (Mean. 74.53%) of the inclusion bodies were located centrally or submarginally and the remaining 14-37.5% (Mean. 25.47%) were situated marginally. Splitter et al. (12) revealed that within erythrocytes 60-70% of *A. ovis* inclusion bodies were located marginally and 30-40% submarginally or centrally. In the case of *A. ovis*, bacterial inclusions were found 35–40% of the time in the central or submarginal part of the erythrocyte of the host, and the remaining 60–65% of the time in the marginal part (20).

Hematological values including packed cell volume, hemoglobin concentration and erythrocyte count in animals infected with *A. ovis* initiated to increase gradually on the day 18-23 post infection until the last day of experiment.

The results of the current experiment indicated that at the early stage of the study, during the days 3-18 post infection, reticulocytes and basophilic stippling were not appeared or presented at very low numbers and there were no changes in MCV and MCHC. Therefore at this stage of the disease anemia was nonregenerative with normocytic normochromic appearance. Thereafter by development of infection reticulocytes gradually increased and reached its maximal level on the day 23 post infection. On the other hand basophilic stippling was detected 9 days post infection and increased significantly up to day 20 of experiment. A significant increased in MCV coincided with highest reticulocytosis. These findings indicated evidence of macrocytic normochromic anemia in the middle stage of experiment. Reticulocytosis, polychromasia and basophilic stippling in blood smears suggested a regenerative anemia in infected animal (21). Naqid et al. (16) reported a macrocytic normochromic anemia in goats naturally infected with *A. ovis*. Anemia of camel anaplasmosis was macrocytic normocromic (22). No significant changes in MCV and MCHC in the goat naturally infected with *A.ovi*s were observed (13).

The hematological findings showed a significant decrease in RBC, Hb, PCV, and MCHC in cattle infected with *A. marginale* (23). No significant changes in MCV value were noted so that, anemia was found to be normocytic hypochromic.

Our experiment showed that there were no significant difference in the means of WBC counts, lymphocytes, neutrophils, eosinophils, and monocytes. Total leukocyte count usually remained unchanged in experimental anaplasmosis (12).

The means of WBC counts, lymphocytes, eosinophils, and monocytes in the infected goat were lower than in the uninfected, but neutrophils counts were higher in the infected animals than in the uninfected animals (13). These differences were not statistically significant. In the study of camel anaplasmosis, (22), a significant rise in total leukocyte count was observed due to increased lymphocyte and decreased nutrophil counts. All the Sahiwal and crossbred cattle infected with *A.maginale* showed significant decreased in WBCs count (24). As molecular evaluation of *Theileria* and *Babesia* spp. was not performed in these studies, the noted changes in WBC total and differential counts might be related to *Babesia* or *Theileria*.

Changes in mean body temperature were not statistically significant, but some of infected animals demonstrated fever during several days post infection. These findings may be related to individual characteristic and defense system of each animal. In experimental anaplasmosis body temperature were usually unchanged, although it increased as high as 41°C in several animals at the peak of parasitaemia (12). Without any evidence of clinical icterus, the mucus membranes were anemic during the days 12-23 post infection. It may be as a result of decrease in PCV, RBC and Hb.

## Conclusion

*Anaplasma ovis* can cause marked anemia that was normocytic normochromic at the beginning of the infection. By the development of the disease and appearance of reticulocytosis, basophilic stippling and polychromasia MCV increased and anemia became macrocytic normochromic. We observed negative correlation between parasitemia and RBC, PCV and Hb values. No significant changes were noticed in the mean of total and differential WBC count. Although some of infected animals showed fever during several days post infection but in most of them body temperature was unchanged. Therefore no significant changes in body temperature were observed. Palness of mucus membranes and anorexia were exhibited during the anemic phase of infection.
